# A rare simultaneous coexistence of epithelioid gastrointestinal stromal tumors and schwannoma in the stomach: a case report

**DOI:** 10.1186/s13000-019-0898-x

**Published:** 2019-10-23

**Authors:** Yuxin Li, Yongliang Teng, Xiaofei Wei, Zhuang Tian, Yuqing Cao, Xiaona Liu, Xiumei Duan

**Affiliations:** Department of Pathology, the First Hospital of Jilin University, Changchun, China

**Keywords:** GISTs, Gastric schwannoma, Case report, PDGFRA, Stomach

## Abstract

**Background:**

Gastrointestinal stromal tumors (GISTs), a type of mesenchymal tumor in the gastrointestinal tract, are believed to be closely associated with PDGFRA and C-KIT mutations. Schwannoma in the stomach, which is an unusual location, is a rare disorder. The simultaneous occurrence of the two diseases is rarer than metachronous occurrences, and its pathological characteristics have not been reported to date. We present a case report on a patient with simultaneous coexistence of gastric schwannoma and GISTs.

**Case presentation:**

A 39-year-old female visited our hospital complaining of intermittent abdominal pain for the previous 3 months. CT revealed a 3.4 cm slight homogeneous enhancement in the lesser curvature of the stomach; the mass was nodular soft tissue, which was removed by radical surgery. Two solid tumors with different volumes were located in the stomach. Histologically and immunohistochemically different, the larger tumor consisted of spindle cells surrounded by a peripheral lymphoid cuff, and was positive for S-100. The larger tumor was therefore classified as a gastric schwannoma. The smaller tumor was composed of medium-sized round, oval cells with amphiphilic granular cytoplasm; vacuolization was also observed. The tumor cells were positive for DOG1 and sporadically positive for CD34 and CD117. Hence, the smaller tumor was diagnosed as epithelioid GISTs. Sanger sequencing revealed that the GIST tumor cells contained a deletion mutation (c.2527_2538 del12,843–846del4), which was located in exon 18 of PDGFRA.

**Conclusion:**

GISTs combined with gastric schwannoma are a considerably rare subgroup of gastric tumors. Related clinical research is comparatively weak, and the mechanism remains unknown. We reviewed related articles to provide knowledge to improve the correct identification, diagnosis and management of patients with gastric cancer. All pathologists involved in the diagnosis and clinicians involved in the treatment should be aware of this new kind of disease pattern to improve their understanding of the disease.

## Background

Gastrointestinal stromal tumors (GISTs), leiomyoma or leiomyosarcoma and gastric schwannoma are tumors of the mesenchymal tissue originating in the stomach; of these, GISTs are the most common mesenchymal tumors in the gastrointestinal tract, accounting for approximately 80% [1, 2]. GISTs can occur in any part of the digestive tract, but the most common location is the stomach (50%~ 60%), followed by the small intestine, colorectal area and esophagus; they rarely occur in the mesenteric, retinal and abdominal cavities [1, 3–7]. GISTs are mainly divided into categories based on morphology: typically a spindle pattern, an epithelioid pattern or a mixed pattern, among which the spindle pattern is the most frequent. Preoperative diagnosis of GISTs is usually established on the basis of computerized tomography (CT) of the abdomen and pelvis or magnetic resonance imaging (MRI). Pathologic diagnosis of GISTs is based on identification of a mesenchymal neoplasm with spindle cell or epithelioid histology. Common histologic features of GISTs include spindle cells with sclerosis matrix and epithelioid cytology in gastric GISTs [4]. Immunohistochemistry is also a significant method for diagnosing GISTs. CD117, DOG1, CD34, Ki-67 and succinate dehydrogenase B (SDHB) are recommended. With the development of precision medicine, molecular identification is becoming more important in the diagnosis of GISTs. GISTs commonly harbor oncogene mutations in the KIT tyrosine kinase, which is a target for the kinase inhibitor imatinib. A subset of GISTs, however, contains mutations in the homologous kinase platelet-derived growth factor receptor alpha (PDGFRA), and the most common of these mutations is resistant to imatinib [3, 4, 7]. GISTs have been reported to coexist with a variety of neoplasms; the percentage of such cases has ranged from 2.95 to 43% [8], but the coexistence of GISTs and gastric schwannoma is rarely found.

## Case presentation

### Clinical history

A 39-year-old female visited our hospital complaining of intermittent abdominal pain for the previous 3 months. The clinical doctor gave her a physical examination: the abdomen was flat, and the abdominal mass was not touched. The clinical diagnosis was stomach swelling and digestive tract hemorrhage. An upper gastrointestinal endoscopy revealed a swollen mass in the gastric antrum and angle. CT revealed a 3.4 cm slight homogeneous enhancement, which was nodular soft tissue in the lesser curvature of the stomach; the body of stomach was poorly filled; the mucosae and serosa were smooth; the definite margin of the tumor was surrounded by fat; and no enlarged lymph node after the abdominal cavity and peritoneum was found. Her disease was diagnosed as gastric tumors. She received laparoscopic gastric resection for gastric lesions.

### Pathological findings

**Fig. 1:**
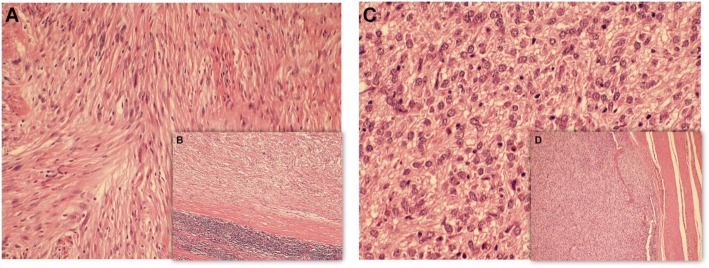
*Histopathological findings*. Hematoxylin-eosin(HE). **a** × 20 objective, **b** × 10 objective) Gastric schwannoma. Spindle cells surrounded by a peripheral lymphoid cuff, which was arranged mainly in small bundles or in a woven pattern. **c** × 40 objective, **d** × 10 objective) GISTs. Medium-sized round, oval cells with amphiphilic cytoplasm that was granular, and vacuolization was observed.The tumor was lamellar, at which the boundary between tumor and normal cells was well demarcated

**Fig. 2:**
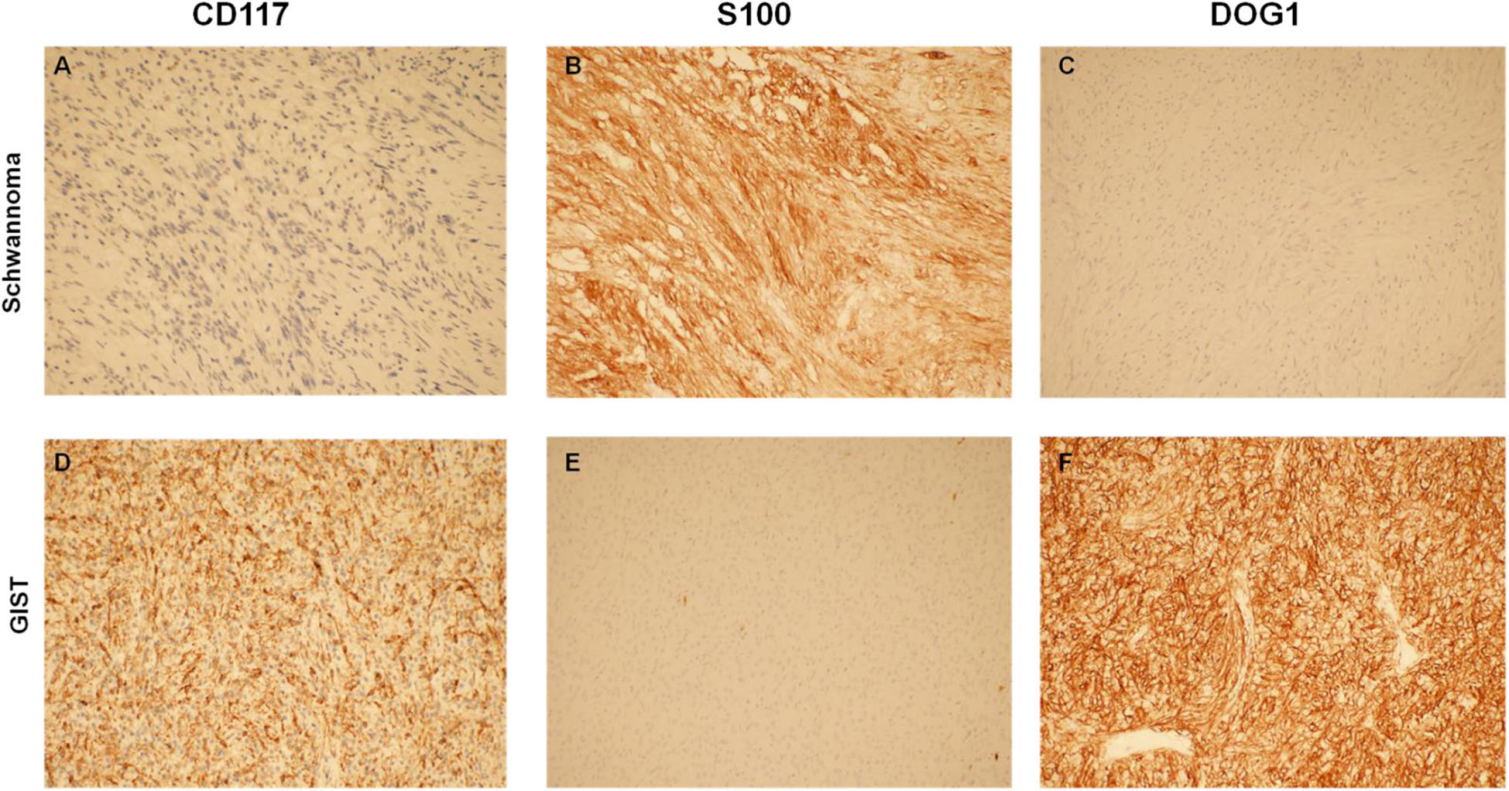
*Histopathological findings.*Immunohistochemical staining. **a**-**c** × 20 objective) Schwannoma. **a**) CD117 negative. **b**) S100 positive. **c**) DOG1 negative. **d**-**e** × 20 objective) GISTs. **d**) CD117 sporadically positive. **e**) S100 negative. **f**) DOG1 postive

According to gross examination, two different solid tumors with different volumes were found in the stomach, measuring 4.3 cm*3.3 cm*2.7 cm and 2.6 cm*2 cm*1.8 cm. Histologically and immunohistochemically, the larger tumor consisted of spindle cells surrounded by a peripheral lymphoid cuff (Fig. 1b), which was arranged mainly in small bundles or in a woven pattern (Fig. 1a). The tumor cells were positive for S-100 (Fig. 2b) and negative for CD117, DOG1 (Fig. 2a, c), CD34, Desmin, smooth muscle actin (SMA) and H-caldesmon (data not shown); the Ki-67 labeling index of the cancer cells was less than 5% (data not shown). The larger tumor was therefore classified as a gastric schwannoma. The smaller one was composed of medium-sized round, oval cells with amphiphilic cytoplasm that was granular, and vacuolization was observed. The tumor was lamellar, at which the boundary between tumor and normal cells was well demarcated (Fig. 1c, d). The tumor cells were positive for DOG1 (Fig. 2f), sporadically positive for CD34 (data not shown) and CD117 (Fig. 2d), and negative for S-100 (Fig. 2e), Desmin, SMA, H-caldesmon, and CK-pan (data not shown). Hence, the smaller tumor was diagnosed with epithelioid GISTs. A total of less than 5 mitoses per 50 high power fields (with a 40 objective) were assessed in most cellular neoplastic areas. The estimation of recurrence risk or death of the GISTs in our case could be considered low risk, depending on tumor size and the mitotic index (Table 1).

**Table 1:** Estimation of the recurrence risk or death linked to the disease in localized and resectable GISTs depending on tumor size and the mitotic index [9]

Risk	Maximum diameter (cm)	Mitotic index (per 50 fields)
Very low risk	< 2	< 5
Low risk	2–5	< 5
Intermediate risk	< 5	6–10
	5–10	< 5
High risk	> 5	> 5
	> 10	All
	All	> 10

**Fig. 3:**
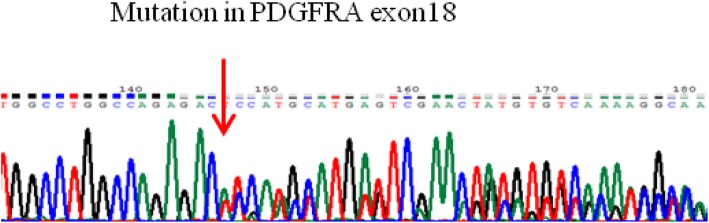
Exon 18 of PDGFRA is deletion mutation type (c.2527_2538 del12,843–846del4) in GISTs

Why did GISTs and gastric schwannoma occur at the same time? Did they have a relationship to the gene mutation? To answer these questions, exons 9, 11, 13, 17 of C-KIT and exons 12 and 18 of PDGFRA were analyzed by PCR and Sanger sequencing both in GISTs and gastric schwannoma. The tumor cells of GISTs contained a deletion mutation (c.2527_2538 del12,843–846del4), which was located in exon 18 of PDGFRA (Fig. 3). However, no gene mutation was detected in gastric schwannoma.

## Discussion

We present a rare case of the simultaneous occurrence of GISTs and gastric schwannoma. Various studies so far have focused on GISTs, so we have unilateral precise recognition in the pathogenesis, diagnosis, and treatment of GIST. However, studies on the mechanisms of the simultaneous occurrence of these two types of tumors are infrequent, as described below.

We believe the most interesting thing about this case is that these two tumors occurred in the stomach at the same time. Highlighting of reports on the specific mechanism of GISTs and gastric schwannoma that occur simultaneously in the stomach is important, due to the remarkably peculiar coexistence, has not been systematically demonstrated. In general, the occurrence of gastric schwannoma alone is already rare. A study by Tao [2] is one of a small number of articles that have a large amount of data on gastric schwannoma. The study showed that 267 patients had gastric GISTs during the same time period—that is, approximately 9 cases of gastric GISTs were observed for each case of gastric schwannoma. Nevertheless, Yang’s case report [10] is the only study that reported the simultaneous occurrence of GISTs and gastric schwannoma. Unfortunately, they did not perform further research on patients with special morphological features, and currently, no systematic study concerning the detection of genetic mutations has been published yet.

However, knowledge about the coexistence of GISTs and other gastrointestinal tumors is increasing, which can provide some inspiration for understanding the new coexisting pattern in our case. Vassos et al. summed various studies on these coexistent diseases and found that the reported frequencies of second malignancies in GIST patients ranged from 2.95 to 43%. Carcinomas of the gastrointestinal tract were the most frequent neoplasms associated with concomitant GISTs; other types include lymphoma, lung or adrenocortical adenoma. The studies also mentioned that most synchronous GISTs were very low/low risk, and this feature also matched the characteristics of our case [8]. Regrettably, little is known about the etiology and pathogenesis of this coexistence. Simple coincidence could be the obvious explanation**,** but various hypotheses have been proposed. Some carcinogens, such as N-methyl-N-nitro-N-nitrosoguanidine, are involved in the development of neoplasms associated with concomitant GISTs in experiments [8]. Theoretically, genetic mutations or combined genetic deregulation could also be associated with the pathogenesis of gastric synchronous tumors. Separately, for our case, in addition to the aforementioned possible causes, we believe the same starting factors of different mesenchymal tumors are involved in the pathogenesis of these two entities because both gastric stromal tumor and gastric schwannoma originate in the mesenchyme.

However, there are many excellent reviews on GISTs alone in the literature dealing with the basic concepts of the pathogenesis of GISTs, which is related to C-KIT and PDGFRA, and the activation of its downstream pathways has been confirmed. The KIT receptor activating mutations occur in 60–96% of GISTs [3–7, 11–13]. Although the mutation rate of PDGFRA is significantly lower than that of C-KIT (3.5%~ 7.2%), it is also one of the mechanisms of the pathogenesis of GISTs [6, 14]. In the analysis of PDGFRA gene mutations, approximately 95% were observed in the stomach, omentum and peritoneum. The most common mutation is located in exon 18, where p.D842V accounts for 60 to 65%, and a few occur in exons 12 and 14 [6, 11–15]. In our case report, the gene mutation of GISTs is the deletion mutation of exon 18 in PDGFRA, which involves deletion of codons 843–846. An interesting report on the investigation of PDGFRA mutations in GISTs was described by Christopher et al. [14]. DIMH842–845/IMH843–846 accounted for 14.9% of the total mutation in PDGFRA. Since codons 843–845 were flanked by aspartic acid (D842 and D846), these two mutations in the protein sequence were equivalent. This is supported by Li Yanyan et al. [13], who performed gene mutation analysis on 827 patients. Their findings revealed that only two cases of DIMH 842–845 and one case of IMH 843–846 were identified as deletion mutations. Therefore, the genetic mutation in our case is relatively scarce in GISTs. A recent review by Corless [14] revealed that deletion mutations of 842–845 and 843–846 of exon 18 in PDGFRA were sensitive to imatinib. In contrast, mutations in the schwannoma of the stomach are infrequent.

The gastric schwannoma and GISTs are from the same mesenchymal tissue of the stomach, which is easily confused and misdiagnosed. Tao et al. [2] showed that the correct preoperative diagnosis rate of schwannoma was only 3.3%, and 21 of 30 patients were misdiagnosed with GISTs. In summary, the identification of these two tumors is of particular importance. The diagnosis of schwannoma is based on immunohistochemical staining of S-100 and vimentin positive and negative results of CD117 and CD34, whereas GISTs are usually positive for CD117, DOG1 and CD34, but S-100 is mostly negative [2, 4, 11, 13]. In addition, CD117 positivity can be found in approximately 95% of C-KIT mutations, but approximately 40% of PDGFRA mutations are weakly positive or negative for CD117 [4, 14, 16]. The positive expression rate of DOG1 in GISTs is up to 94.8%, and it has a high sensitivity of 89%, and its positive rate in epithelioid GISTs is higher than that in CD117 [4, 12, 17].

## Conclusion

We describe the first known case report on the coexistence of gastric schwannoma with epithelioid GISTs using genetics, immunochemistry and histology at our institute. These rare and unique coexistent tumors could possibly be misdiagnosed preoperatively, especially when the diagnosis is based solely on CT without histological and immunohistochemical analyses. Furthermore, we have observed this phenomenon from the perspective of genetics, which could provide a potential kind of pathogenesis and a new pattern of co-occurrence for neoplasms associated with concomitant GISTs. The concurrence of other malignancies with GISTs raises discussion about a potential common origin and carcinogenetic mechanisms in these distinct tumor types; thus, a case series study would be needed. Finally, we expect that our case report would contribute to the recognition of morphological and biological characteristics of these coexistent diseases, which support the precise diagnosis and correct interpretation to avoid inappropriate under- or overtreatment of the patients in the future.

## Data Availability

The datasets used and/or analyzed during the current study are available from the corresponding author upon reasonable request.

## Abbreviations

CT: Computerized tomography
GISTs: Gastrointestinal stromal tumors
MRI: Magnetic resonance imaging
PDGFRA: Platelet-derived growth factor receptor alpha
SDHB: Succinate dehydrogenase B
SMA: Smooth muscle actin
